# Supplementary material for: Modeling glioblastoma heterogeneity as a dynamic network of cell states
Source: Mol Syst Biol. 2021 Sep 16;17(9):e10105. doi: 10.15252/msb.202010105 (PMC8444284; doi:10.15252/msb.202010105)
Supplement: Supplementary file 5 — Source Data for Figure 3 [file MSB-17-e10105-s001.zip › Figure3A_sourcedata/GSEA_3065/hallmarks_state5.GseaPreranked.1623416302988/HALLMARK_GLYCOLYSIS.html]

Details for gene set HALLMARK\_GLYCOLYSIS[GSEA]

|  || Dataset | state5 |
| Phenotype | NoPhenotypeAvailable |
| Upregulated in class | na\_neg |
| GeneSet | HALLMARK\_GLYCOLYSIS |
| Enrichment Score (ES) | -0.3079499 |
| Normalized Enrichment Score (NES) | -1.1075124 |
| Nominal p-value | 0.25997582 |
| FDR q-value | 0.56360704 |
| FWER p-Value | 1.0 |
Table: GSEA Results Summary

  

Fig 1: Enrichment plot: HALLMARK\_GLYCOLYSIS      
 Profile of the Running ES Score & Positions of GeneSet Members on the Rank Ordered List

  

| PROBE | GENE SYMBOL | GENE\_TITLE | RANK IN GENE LIST | RANK METRIC SCORE | RUNNING ES | CORE ENRICHMENT || 1 | HSPA5 |  |  | 43 | 0.698 | 0.0203 | No |
| 2 | GPC1 |  |  | 98 | 0.530 | 0.0333 | No |
| 3 | GUSB |  |  | 143 | 0.489 | 0.0460 | No |
| 4 | VCAN |  |  | 191 | 0.444 | 0.0567 | No |
| 5 | CHST12 |  |  | 206 | 0.436 | 0.0709 | No |
| 6 | AGRN |  |  | 271 | 0.398 | 0.0780 | No |
| 7 | CD44 |  |  | 378 | 0.349 | 0.0786 | No |
| 8 | CXCR4 |  |  | 450 | 0.325 | 0.0823 | No |
| 9 | STC1 |  |  | 530 | 0.303 | 0.0843 | No |
| 10 | PLOD2 |  |  | 613 | 0.282 | 0.0852 | No |
| 11 | ZNF292 |  |  | 656 | 0.274 | 0.0903 | No |
| 12 | SDC3 |  |  | 728 | 0.255 | 0.0915 | No |
| 13 | P4HA1 |  |  | 792 | 0.242 | 0.0931 | No |
| 14 | IDH1 |  |  | 793 | 0.242 | 0.1018 | No |
| 15 | NT5E |  |  | 834 | 0.235 | 0.1058 | No |
| 16 | SLC35A3 |  |  | 841 | 0.234 | 0.1135 | No |
| 17 | PAM |  |  | 844 | 0.233 | 0.1217 | No |
| 18 | ANKZF1 |  |  | 879 | 0.228 | 0.1261 | No |
| 19 | CHPF2 |  |  | 916 | 0.222 | 0.1301 | No |
| 20 | B4GALT4 |  |  | 1037 | 0.206 | 0.1239 | No |
| 21 | EXT2 |  |  | 1090 | 0.199 | 0.1252 | No |
| 22 | QSOX1 |  |  | 1125 | 0.195 | 0.1284 | No |
| 23 | TGFBI |  |  | 1177 | 0.188 | 0.1294 | No |
| 24 | NASP |  |  | 1225 | 0.182 | 0.1307 | No |
| 25 | B3GAT1 |  |  | 1261 | 0.177 | 0.1331 | No |
| 26 | GPC4 |  |  | 1288 | 0.173 | 0.1364 | No |
| 27 | CLN6 |  |  | 1301 | 0.171 | 0.1412 | No |
| 28 | COL5A1 |  |  | 1328 | 0.168 | 0.1444 | No |
| 29 | PAXIP1 |  |  | 1390 | 0.162 | 0.1433 | No |
| 30 | SDC1 |  |  | 1464 | 0.156 | 0.1407 | No |
| 31 | TPST1 |  |  | 1486 | 0.154 | 0.1439 | No |
| 32 | EGFR |  |  | 1547 | 0.147 | 0.1424 | No |
| 33 | CHPF |  |  | 1616 | 0.140 | 0.1398 | No |
| 34 | VEGFA |  |  | 1634 | 0.139 | 0.1429 | No |
| 35 | SLC25A13 |  |  | 1707 | 0.133 | 0.1395 | No |
| 36 | HS2ST1 |  |  | 1744 | 0.130 | 0.1401 | No |
| 37 | ERO1A |  |  | 1804 | 0.127 | 0.1380 | No |
| 38 | PMM2 |  |  | 1868 | 0.121 | 0.1353 | No |
| 39 | PLOD1 |  |  | 1919 | 0.117 | 0.1338 | No |
| 40 | KDELR3 |  |  | 1926 | 0.117 | 0.1374 | No |
| 41 | SRD5A3 |  |  | 1961 | 0.114 | 0.1377 | No |
| 42 | MET |  |  | 1992 | 0.112 | 0.1383 | No |
| 43 | IL13RA1 |  |  | 2016 | 0.110 | 0.1397 | No |
| 44 | P4HA2 |  |  | 2022 | 0.110 | 0.1431 | No |
| 45 | ALG1 |  |  | 2026 | 0.110 | 0.1467 | No |
| 46 | IGFBP3 |  |  | 2037 | 0.109 | 0.1495 | No |
| 47 | B3GAT3 |  |  | 2097 | 0.106 | 0.1466 | No |
| 48 | FUT8 |  |  | 2157 | 0.101 | 0.1436 | No |
| 49 | ME2 |  |  | 2206 | 0.098 | 0.1417 | No |
| 50 | B4GALT1 |  |  | 2347 | 0.089 | 0.1291 | No |
| 51 | SLC37A4 |  |  | 2484 | 0.081 | 0.1166 | No |
| 52 | ADORA2B |  |  | 2563 | 0.076 | 0.1105 | No |
| 53 | CAPN5 |  |  | 2765 | 0.065 | 0.0901 | No |
| 54 | B3GALT6 |  |  | 2870 | 0.059 | 0.0805 | No |
| 55 | GLCE |  |  | 3285 | 0.037 | 0.0351 | No |
| 56 | STC2 |  |  | 3306 | 0.036 | 0.0341 | No |
| 57 | GALE |  |  | 3486 | 0.028 | 0.0149 | No |
| 58 | ECD |  |  | 3500 | 0.027 | 0.0144 | No |
| 59 | AGL |  |  | 3509 | 0.027 | 0.0144 | No |
| 60 | NSDHL |  |  | 3526 | 0.026 | 0.0136 | No |
| 61 | COG2 |  |  | 3569 | 0.024 | 0.0097 | No |
| 62 | MED24 |  |  | 3623 | 0.023 | 0.0045 | No |
| 63 | BPNT1 |  |  | 3665 | 0.021 | 0.0007 | No |
| 64 | MXI1 |  |  | 3791 | 0.016 | -0.0129 | No |
| 65 | VLDLR |  |  | 3854 | 0.014 | -0.0194 | No |
| 66 | HOMER1 |  |  | 4062 | 0.005 | -0.0427 | No |
| 67 | TPBG |  |  | 4075 | 0.004 | -0.0439 | No |
| 68 | ALDH7A1 |  |  | 4221 | -0.002 | -0.0602 | No |
| 69 | MPI |  |  | 4298 | -0.006 | -0.0686 | No |
| 70 | RPE |  |  | 4403 | -0.010 | -0.0800 | No |
| 71 | GMPPB |  |  | 4504 | -0.014 | -0.0908 | No |
| 72 | AK3 |  |  | 4543 | -0.016 | -0.0945 | No |
| 73 | SDC2 |  |  | 4596 | -0.018 | -0.0997 | No |
| 74 | CDK1 |  |  | 4766 | -0.025 | -0.1179 | No |
| 75 | HMMR |  |  | 4826 | -0.027 | -0.1236 | No |
| 76 | AKR1A1 |  |  | 4870 | -0.028 | -0.1275 | No |
| 77 | GFPT1 |  |  | 4876 | -0.028 | -0.1270 | No |
| 78 | PGM2 |  |  | 4885 | -0.029 | -0.1269 | No |
| 79 | UGP2 |  |  | 5013 | -0.034 | -0.1401 | No |
| 80 | GMPPA |  |  | 5082 | -0.036 | -0.1464 | No |
| 81 | GOT2 |  |  | 5108 | -0.037 | -0.1479 | No |
| 82 | COPB2 |  |  | 5597 | -0.057 | -0.2010 | No |
| 83 | B4GALT7 |  |  | 5626 | -0.059 | -0.2021 | No |
| 84 | ISG20 |  |  | 5757 | -0.064 | -0.2145 | No |
| 85 | SLC25A10 |  |  | 5851 | -0.067 | -0.2225 | No |
| 86 | PYGB |  |  | 5874 | -0.069 | -0.2226 | No |
| 87 | ENO2 |  |  | 5895 | -0.069 | -0.2223 | No |
| 88 | ALDOA |  |  | 5909 | -0.070 | -0.2213 | No |
| 89 | ALDH9A1 |  |  | 6006 | -0.074 | -0.2294 | No |
| 90 | IRS2 |  |  | 6033 | -0.076 | -0.2296 | No |
| 91 | SDHC |  |  | 6193 | -0.085 | -0.2446 | No |
| 92 | CENPA |  |  | 6374 | -0.094 | -0.2615 | No |
| 93 | NOL3 |  |  | 6417 | -0.096 | -0.2628 | No |
| 94 | PYGL |  |  | 6492 | -0.101 | -0.2675 | No |
| 95 | DPYSL4 |  |  | 6520 | -0.102 | -0.2669 | No |
| 96 | CHST2 |  |  | 6560 | -0.106 | -0.2675 | No |
| 97 | DEPDC1 |  |  | 6723 | -0.115 | -0.2817 | No |
| 98 | NDUFV3 |  |  | 6726 | -0.115 | -0.2777 | No |
| 99 | GOT1 |  |  | 6759 | -0.118 | -0.2771 | No |
| 100 | PRPS1 |  |  | 6774 | -0.119 | -0.2744 | No |
| 101 | GLRX |  |  | 6822 | -0.123 | -0.2753 | No |
| 102 | PFKP |  |  | 6845 | -0.124 | -0.2733 | No |
| 103 | DDIT4 |  |  | 6968 | -0.135 | -0.2822 | No |
| 104 | AURKA |  |  | 6990 | -0.136 | -0.2797 | No |
| 105 | KIF2A |  |  | 7030 | -0.139 | -0.2791 | No |
| 106 | CITED2 |  |  | 7120 | -0.147 | -0.2838 | No |
| 107 | HAX1 |  |  | 7183 | -0.154 | -0.2853 | No |
| 108 | CASP6 |  |  | 7206 | -0.156 | -0.2822 | No |
| 109 | RRAGD |  |  | 7321 | -0.170 | -0.2889 | No |
| 110 | FKBP4 |  |  | 7326 | -0.170 | -0.2832 | No |
| 111 | DLD |  |  | 7363 | -0.176 | -0.2809 | No |
| 112 | MDH1 |  |  | 7422 | -0.184 | -0.2809 | No |
| 113 | RBCK1 |  |  | 7431 | -0.185 | -0.2751 | No |
| 114 | GALK1 |  |  | 7610 | -0.206 | -0.2878 | No |
| 115 | B4GALT2 |  |  | 7697 | -0.218 | -0.2897 | No |
| 116 | ARPP19 |  |  | 7811 | -0.236 | -0.2939 | No |
| 117 | SAP30 |  |  | 7936 | -0.263 | -0.2985 | Yes |
| 118 | GNPDA1 |  |  | 7975 | -0.271 | -0.2930 | Yes |
| 119 | FAM162A |  |  | 7983 | -0.272 | -0.2840 | Yes |
| 120 | CYB5A |  |  | 8086 | -0.294 | -0.2849 | Yes |
| 121 | POLR3K |  |  | 8132 | -0.305 | -0.2790 | Yes |
| 122 | PSMC4 |  |  | 8153 | -0.309 | -0.2701 | Yes |
| 123 | LDHA |  |  | 8263 | -0.343 | -0.2701 | Yes |
| 124 | G6PD |  |  | 8271 | -0.346 | -0.2584 | Yes |
| 125 | MIF |  |  | 8296 | -0.353 | -0.2484 | Yes |
| 126 | SOX9 |  |  | 8327 | -0.361 | -0.2387 | Yes |
| 127 | PPP2CB |  |  | 8347 | -0.369 | -0.2276 | Yes |
| 128 | PGLS |  |  | 8357 | -0.372 | -0.2152 | Yes |
| 129 | TALDO1 |  |  | 8393 | -0.386 | -0.2052 | Yes |
| 130 | PGK1 |  |  | 8452 | -0.408 | -0.1970 | Yes |
| 131 | PGAM1 |  |  | 8698 | -0.558 | -0.2046 | Yes |
| 132 | HDLBP |  |  | 8699 | -0.560 | -0.1844 | Yes |
| 133 | MDH2 |  |  | 8733 | -0.593 | -0.1668 | Yes |
| 134 | ENO1 |  |  | 8734 | -0.593 | -0.1454 | Yes |
| 135 | STMN1 |  |  | 8783 | -0.638 | -0.1278 | Yes |
| 136 | SOD1 |  |  | 8848 | -0.709 | -0.1095 | Yes |
| 137 | PPIA |  |  | 8913 | -0.797 | -0.0880 | Yes |
| 138 | TPI1 |  |  | 8939 | -0.852 | -0.0601 | Yes |
| 139 | TXN |  |  | 8952 | -0.885 | -0.0295 | Yes |
| 140 | PKM |  |  | 8959 | -0.915 | 0.0028 | Yes |
Table: GSEA details [plain text format]

  

Fig 2: HALLMARK\_GLYCOLYSIS: Random ES distribution      
 Gene set null distribution of ES for **HALLMARK\_GLYCOLYSIS**
